# Comparing biofeedback device vs diaphragmatic breathing for bloating relief: A randomized controlled trial

**DOI:** 10.1515/med-2025-1319

**Published:** 2025-11-15

**Authors:** Subhankar Chakraborty

**Affiliations:** Department of Gastroenterology, Hepatology and Nutrition, The Ohio State University, 395 W 12th Avenue, Columbus, Ohio, 43016, United States

**Keywords:** bloating, breathing exercise, diaphragmatic breathing, biofeedback, anxiety, depression, gastrointestinal

## Abstract

**Background:**

Abdominal bloating is a very common and often quite debilitating complaint affecting nearly 18% of people worldwide. There is very little known about whether or not breathing exercises can help reduce bloating.

**Methods:**

In a prospective study, we investigated the effects of breathing exercises using either a PDF of written instructions on diaphragmatic breathing or a biofeedback respiratory practice device (Calmigo^®^) on abdominal bloating. Participants were randomized to either intervention and asked to practice three times daily for 3 min each time for 6 weeks. Bloating was evaluated at baseline and every week for 7 weeks via online self-reported surveys.

**Results:**

Eighty-five participants were randomized to either written instructions (*n* = 36) or Calmigo^®^ (*n* = 45). There was no difference between the two groups at baseline in demographic features, anxiety, depression, or bloating severity. While bloating improved at all time points, it was statistically significant in the 4 weeks in the Calmigo^®^ group and at weeks 3, 4, and 5 in the written instructions group. Higher baseline anxiety and lower depression symptoms were predictive of improvement in bloating at 4 weeks.

**Conclusion:**

Brief breathing exercises, either using written instructions or Calmigo^®^, improve bloating symptoms. These could be considered in the management of functional abdominal bloating.

## 1 Introduction

Abdominal bloating is a common gastrointestinal problem. The symptoms are often debilitating and affect a person’s quality of life. While bloating can be caused by many things, including constipation, obesity, and gastrointestinal dysmotility, the most prevalent type is functional bloating, where there is no organic cause identified to explain the symptoms. A study from the Rome Foundation reported that approximately 1 in 5 people worldwide suffer from bloating. Bloating is more common in women and decreases as we age [1]. A recent clinical update from the American Gastroenterology Association highlighted the clinical significance and challenges of treating bloating and suggested using diaphragmatic breathing exercises to treat patients [2]. However, the expert review only had two studies on the role of breathing exercises in alleviating bloating, highlighting the paucity of data on the effects of these exercises.

Given the lack of data on the effects of breathing exercises on bloating, we performed this prospective study where we compared written instructions on how to do diaphragmatic breathing with a handheld respiratory biofeedback device (Calmigo^®^) to explore their effects on bloating.

## 2 Methods

This was a prospective study conducted at a tertiary university in the Midwestern United States. Participants were enrolled from across the United States. The protocol was approved by the Institutional Review Board at our University (Study ID: 2022H0256). To be eligible, participants had to be at least 18 years of age and understand English. Further, they should have at least a moderately severe degree of one of the following GI symptoms: bloating, nausea, early satiety, *post-prandial* fullness, or abdominal pain as assessed by a screening questionnaire. Additionally, they should have been experiencing at least moderate anxiety (HADS-anxiety score of 11 or more) or depression symptoms (HADS-Depression score 11 or more) as measured by the Hospital Anxiety and Depression questionnaire (HADS). We selected these criteria for several reasons: a) a greater possibility of detecting a change in those who are suffering from more severe symptoms, and b) there is evidence from the medical literature that patients with higher levels of anxiety or depression experience greater benefit from breathing exercises, particularly in the context of disorders of gut–brain interaction. A meta-analysis of randomized controlled trials found that a reduction in anxiety and depressive symptoms with breathwork interventions was associated with baseline severity of these symptoms, suggesting that individuals with higher baseline anxiety or depression may derive meaningful benefit from these interventions [3]. Further, a real-world analysis of digital therapeutic tools to alleviate depression and anxiety demonstrated that engagement with breathing exercises was associated with significant reduction in anxiety, and that this effect was most pronounced in users with moderate baseline symptoms [4].

Information about the study was either shared with patients seen in our GI motility clinic or through two resources available to us through the University: Study Search (https://wexnermedical.osu.edu/participate-in-research) and Research Match (https://www.researchmatch.org/?route=ohsu). Those who were interested completed two qualifying surveys that enquired about the severity of bloating and symptoms of anxiety and depression. Participants were asked to rate abdominal bloating, defined as a feeling that they needed to loosen their clothes on a scale from 0 (None), 1 (Mild), 2 (Moderate), 3 (Severe), and 4 (Very severe) [5]. Mood was assessed using the HADS Questionnaire, a widely used tool in medical research [6]. Eligible individuals were sent the consent form to review. Informed consent was obtained electronically using RedCap.

After enrollment, participants completed baseline surveys about demographics, after which they were randomized to either the device arm (Calmigo^®^) or to written instructions arm. The randomization was based on their age, sex, BMI, and diabetes history. We created the randomization Excel file using the Clinical Trial Randomization Tool available free from the National Cancer Institute (https://ctrandomization.cancer.gov/tool/). Those randomized to the written instructions for Diaphragmatic breathing (DB) arm were emailed a PDF containing instructions on how to perform these exercises, along with a brief description about the diaphragm and how diaphragmatic breathing works to reduce intra-abdominal pressure (Appendix 1). Those who were randomized to the Calmigo^®^ group were mailed their device. The package included instructions on how to use the device and also a QR code where they could watch a video on how to use it. Once they received the device, they were recommended to complete a brief training call with Calmigo^®^ to ensure they knew how to properly use it. Calmigo^®^ is a battery-powered general wellness device intended to ease feelings of stress or anxiousness. When a user exhales into the device, it provides adaptive biofeedback – light, sound, and vibration – indicating the target exhalation duration. An onboard algorithm sets the target from the user’s recent breathing and gradually lengthens it while remaining within the user’s capability; it does not control exhalation force. The device includes an optional solid scent element (enabled in the study unless the patient declined its use due to personal preference) [7]. Both groups were asked to practice breathing exercises thrice a day for 3 min at a time for 6 weeks. They were advised to find a quiet place, turn off distractions, and preferably practice at the same time every day. They were also advised to practice either lying down or sitting to avoid feeling dizzy while breathing. The duration and frequency of breathing exercises in our study were chosen based on a previous study using Calmigo^®^ that had used the same duration and frequency and demonstrated that Calmigo^®^ use can reduce anxiety in test-takers [7]. We chose a 6-week period as most studies on GI interventions are usually done for about 6–8 weeks to ensure adequate time to see an effect of the intervention [8,9].

At the end of each week, participants received an electronic survey to complete, wherein they were asked to rate the severity of bloating. The final survey was sent to participants 1 week after finishing the 6 weeks of exercises (week 7 of the intervention period). The primary outcome was improvement in bloating at the 4 week mark by at least 1 unit from baseline. Those who achieved this outcome were deemed “responders.” Those whose symptom remained unchanged or worsened compared to baseline were deemed “non-responders.”

All data were entered into REDCap, which was also used to send surveys to participants (https://redcap.osumc.edu/redcap/index.php). De-identified data were analyzed using the Data Analyzer App (https://data-analysis-master.replit.app/). We conducted a modified intention-to-treat analysis as we included those who completed the baseline surveys in our analysis. Continuous data were expressed as an average, with standard deviation used to assess variation. Categorical data were expressed as frequency and percentage. Continuous data were compared between groups using an independent *t*-test (if there were 2 groups) or one-way ANOVA (if >2 groups). Paired *t*-tests were used to compare the change in bloating severity at a given week compared to that at baseline. Bonferroni correction was applied for multiple comparisons for the paired *t*-test due to the exploratory nature of our analysis. Categorical data were compared using the chi-square test. Proportions were compared using the Fisher’s exact *t*-test. All results were expressed with a 95% confidence interval where applicable. *p*-Values were provided for all statistical tests. A *p*-value of less than 0.05 was considered statistically significant.


**Ethical approval:** Ethical approval was obtained from The Ohio State University Institutional Review Board (Study ID: 2022H0256).
**Informed consent:** Informed consent was obtained electronically from all participants prior to enrollment.

## 3 Results

**Figure 1:**
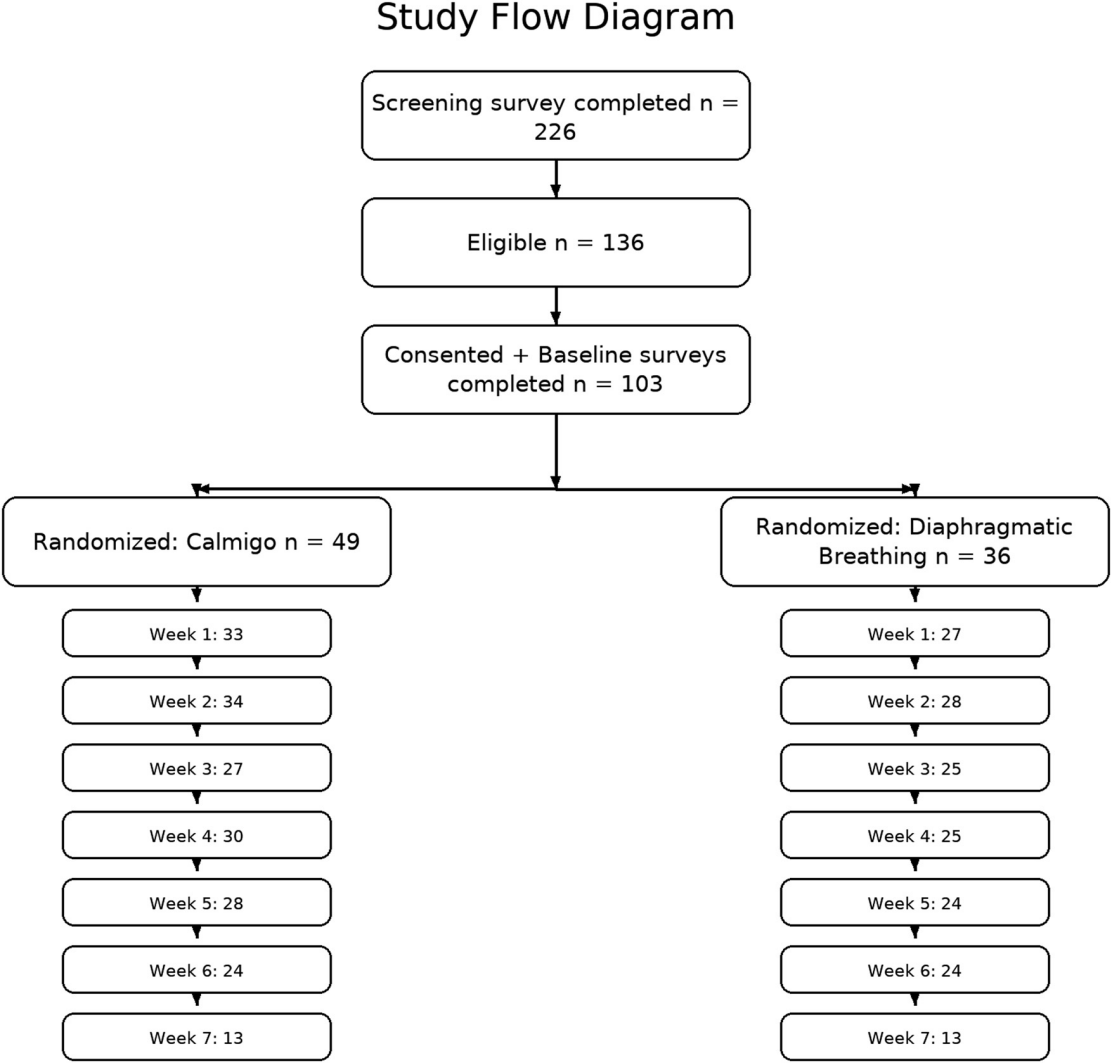
Flow diagram illustrating participant progression through the study. A total of 225 individuals completed the screening survey, of which 136 were eligible. Of these, 103 participants completed the consent form, and 85 completed baseline surveys and were eligible for randomization. Participants were randomized into two groups: 49 to the Calmigo^®^ intervention and 36 to the diaphragmatic breathing (written instructions) group. Weekly survey completion rates are shown for each group.

Figure 1 shows the participant enrollment and progression through the study; 226 people completed the screening surveys. Of the 136 who were eligible, 103 completed the consent form, and 85 of them completed the baseline surveys. They were randomized to either Calmigo^®^ (*n* = 49) or written instructions for DB exercises (*n* = 36).

### 3.1 Baseline characteristics

There was no difference in demographic characteristics like age (*p* = 0.41), race (*p* = 0.67), sex (*p* = 0.17), or body mass index (BMI, *p* = 0.60) between the DB and Calmigo^®^ groups. There was also no difference in HADS-anxiety (*p* = 0.72) or depression (*p* = 0.24) or in the proportion of diabetes (*p* = 0.16). The two groups were similar in the baseline severity of bloating (*p* = 0.93), as shown in Table 1.

**Table 1:** Comparison of baseline characteristics between Calmigo^®^ and written instructions for diaphragmatic breathing group

	Calmigo^®^ (*N* = 49)	Diaphragmatic breathing (*N* = 36)	*p*-value
	Mean (SD)	Mean (SD)	
Age	41.08 (13.89)	43.47 (12.14)	0.411
Race = White	47	32	0.675
Sex = Female	39	32	0.169
BMI	28.37 (9.07)	27.35 (8.05)	0.601
HADS-anxiety	14.15 (2.92)	13.89 (3.56)	0.717
HADS-depression	9.89 (4.55)	11.03 (4.03)	0.240
Diabetes = No	40	34	0.158
Baseline bloating severity	2.41 (1.00)	2.39 (1.08)	0.93

#### 3.1.1 Bloating

**Figure 2:**
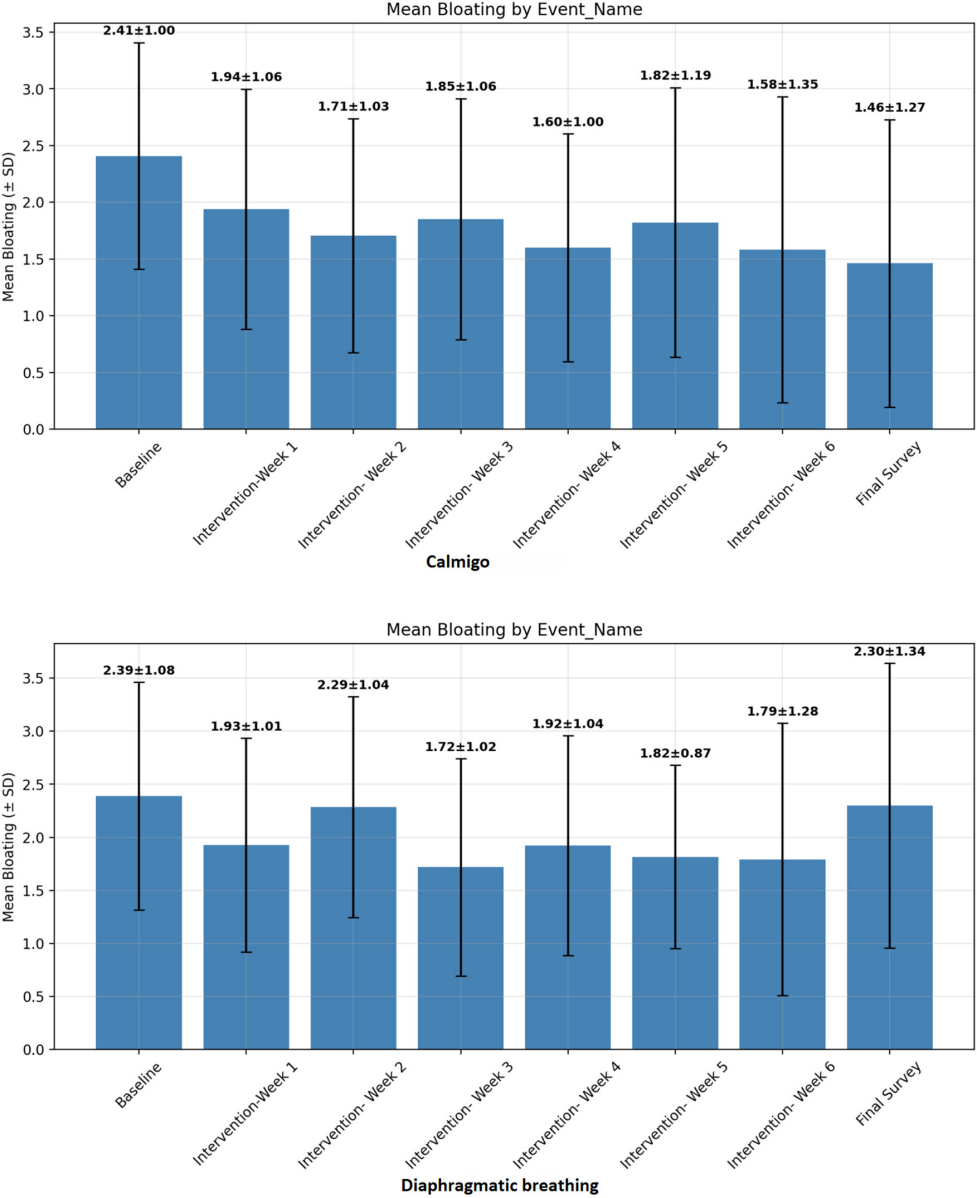
Mean severity of bloating at baseline and during intervention. Top panel: Calmigo^®^; and bottom panel: diaphragmatic breathing instructions. The bars represent the standard deviation.

The mean intensity of bloating each week is shown in the bar graph in Figure 2. Changes in subjective severity of bloating from baseline over the 7-week intervention period are summarized in Table 2. For each week, the number of participants with available data both at baseline and the respective time point, mean change from baseline, standardized effect size, and *p*-value for paired *t*-test are reported.

**Table 2:** Change in bloating severity from baseline with breathing intervention

Time	N	Change from baseline	Effect size	Paired *t*-test *p*-value	Significant after Bonferroni correction (Y/N)	*p*-value Calmigo^®^ vs DB
**Calmigo** ^®^						
Δ Week 1	33	−0.364	−0.276	0.123	No	0.017
Δ Week 2	34	−0.618	−0.547	0.003	No	0.254
Δ Week 3	27	−0.407	−0.376	0.062	No	0.154
Δ Week 4	30	−0.700	−0.709	0.001	**Yes**	0.521
Δ Week 5	28	−0.500	−0.427	0.032	No	0.321
Δ Week 6	24	−0.667	−0.497	0.023	No	0.588
Δ Week 7	13	−0.846	−0.629	0.043	No	0.249
**Diaphragmatic breathing (DB)**						
Δ Week 1	27	−0.481	−0.515	0.013	No	
Δ Week 2	28	−0.429	−0.466	0.020	No	
Δ Week 3	25	−0.720	−0.705	0.002	**Yes**	
Δ Week 4	25	−0.560	−0.787	0.001	**Yes**	
Δ Week 5	24	−0.667	−0.950	<0.001	**Yes**	
Δ Week 6	24	−0.500	−0.565	0.011	No	
Δ Week 7	13	−0.308	−0.325	0.264	No	

In the Calmigo^®^ group, a reduction in bloating was observed throughout the study period, but a statistically significant decrease was only detected at Week 4 after Bonferroni correction (*p* = 0.001), with a medium effect size (−0.709).

In the DB group, bloating decreased at all time points; however, only Weeks 3–5 remained statistically significant after Bonferroni correction (*p* = 0.002, 0.001, and <0.001, respectively). Effect sizes were medium to large (−0.71 to −0.95).

There was no difference in the degree of change in bloating between the Calmigo^®^ and DB groups at matching time points (Table 2)

### 3.2 Influence of baseline characteristics on improvement in bloating

To understand whether baseline characteristics could distinguish between those who had an improvement in bloating (responders) from those in whom symptoms were unchanged or worse (non-responders) at the end of 4 weeks, we compared demographic characteristics, mood, and baseline severity of bloating between responders and non-responders. As both groups had significant improvement from baseline at week 4, we chose to examine predictors of response at week 4.

#### 3.2.1 Calmigo^®^

In the Calmigo^®^ arm, no significant differences were observed between Week-4 responders and non-responders in age, BMI, baseline bloating severity, HADS-Anxiety, or HADS-Depression (Table 3). Race (White), sex (female), and history of diabetes were also similar between groups (*p* = 1.00, 0.056, and 1.00, respectively).

**Table 3:** Comparison of participant characteristics based on improvement after Calmigo^®^ use for 4 weeks

	Responders (*n* = 20)	Non-responders (*n* = 10)	*p*-value	Effect size
Age	43.35 ± 14.58	41.50 ± 15.42	0.750	0.129
BMI	25.80 ± 6.79	25.48 ± 7.44	0.906	0.048
Baseline bloating severity	2.30 ± 0.80	2.30 ± 0.82	1.00	0.00
HADS-anxiety	13.95 ± 3.00	13.90 ± 2.56	0.964	0.018
HADS-depression	9.20 ± 4.29	11.80 ± 4.78	0.142	−0.605

#### 3.2.2 Diaphragmatic breathing

No significant differences were observed between Week-4 responders and non-responders in age, BMI, baseline bloating severity, HADS-Anxiety, or HADS-Depression (Table 4). The proportions of White race, female sex, and diabetes history were likewise similar (*p* = 1.00, 0.16, and 1.00, respectively).

**Table 4:** Comparison of participant characteristics based on improvement after Diaphragmatic breathing exercises for 4 weeks

	Responders (*n* = 13)	Non-responders (*n* = 12)	*p*-value	Effect size
Age	42.85 ± 11.82	42.33 ± 11.70	0.914	0.045
BMI	25.19 ± 6.70	27.30 ± 10.49	0.552	−0.252
Baseline bloating severity	2.69 ± 1.03	1.92 ± 1.38	0.123	0.669
HADS-anxiety	14.92 ± 2.90	12.75 ± 4.14	0.139	0.639
HADS-depression	11.23 ± 3.54	10.17 ± 3.78	0.474	0.303

On univariate regression, a higher baseline HADS-Anxiety score (*p* < 0.0001) and a lower baseline HADS-Depression score (*p* = 0.027) was associated with a greater likelihood of improvement in bloating after 4 weeks. Demographics, history of diabetes, baseline bloating severity, and intervention type (DB vs Calmigo^®^) did not significantly predict improvement (Table 5).

**Table 5:** Predictors of improvement in bloating after 4 weeks

	Odds ratio	95% C.I.	*p*-value
Age	1.007	0.99–1.02	0.257
Race = White	0.886	0.509–1.542	0.669
Sex = Male	0.273	0.053–1.408	0.121
BMI	0.984	0.97–1.004	0.122
Diabetes = Yes	1.00	0.568–1.760	0.998
Baseline bloating severity	1.44	0.83–2.51	0.20
HADS-anxiety	1.116	1.074–1.159	<0.0001
HADS-depression	0.947	0.902–0.994	0.027
Group = Diaphragmatic breathing	0.626	0.286–1.372	0.242

## 4 Discussion

This is the first study comparing the effect of two different breathing interventions- written instructions for DB and device-guided biofeedback (Calmigo^®^) on the severity of abdominal bloating. Our results show that both interventions decreased bloating. While a reduction was observed in all weeks, this was statistically significant during weeks 3–5 for DB and during week 4 for Calmigo^®^. Those with higher baseline anxiety and lower baseline depression symptoms were more likely to improve with breathing exercises. Improvement was not impacted by the type of breathing intervention.

Studies examining the effect of breathing exercises on abdominal bloating are limited. In one open-label study, guided diaphragmatic breathing instruction combined with bloating-targeted hypnotic suggestions improved severity of bloating after 7 weeks in 23 people with functional bowel disorders and bloating as a predominant symptom [10]. Slow deep breathing (six breaths per minute with 4 s inhalation and 6 s exhalation) practiced for 30 min 5 days a week led to improvement in symptoms of irritable bowel syndrome, stool consistency, stool frequency, and decreased rectal sensitivity as measured by anorectal manometry [11]. A placebo-controlled trial comparing biofeedback therapy to a control group in patients with post-meal abdominal distension found a significant reduction in abdominal distension scores in the biofeedback group but not in the placebo [12]. The mechanism by which breathing exercises reduce bloating is not well understood. However, a study in patients with functional abdominal distension found that the episodes of increased distension were associated with contraction of the diaphragm and intercostal muscles, an increase in lung volume, and protrusion of the anterior abdominal wall. Biofeedback therapy reduced intercostal and diaphragm activity and activated the internal oblique muscle with a reduction in abdominal girth [13].

Brief (10 min) *post-prandial* DB decreased the number of reflux episodes in patients with proven GERD, suggesting a mechanism by which it could reduce symptoms of GERD [14]. Another study in patients with GERD refractory to proton pump inhibitors and belching symptoms found that 4 weekly sessions of DB training, each 30 min long, reduced subjective belching by at least 50% in 15 patients. This was associated with a reduction in GERD symptoms and improvement in quality-of-life scores. Further, these changes were sustained for 4 months after DB treatment [15]. The mechanism here appears to be improvement in the tone of the gastroesophageal junction and restoration of a negative gastroesophageal pressure gradient [16].

The Calmigo^®^ device has previously been reported to improve symptoms of anxiety among students. A randomized controlled trial in university students with test anxiety compared Calmigo^®^ with self-directed breathing exercises and psychoeducation during the exam period (*N* = 34). Calmigo^®^ group showed a significant reduction in test-anxiety symptoms from pre- to post-intervention; the device group also reported decreases in depression and anxiety symptoms and higher psychological well-being [7]. The likely mechanism behind Calmigo^®^’s effect includes modulation of autonomic arousal and visceral perception, processes that have also been suggested to be involved in bloating and abdominal distension. Although conducted in a non-GI population, this trial suggested that Calmigo^®^ was better than instructions alone. We however did not observe any differences between Calmigo^®^ and diaphragmatic breathing in our study.

Brief breathing exercises have been investigated in other conditions too. A single 15-min mindful attention to breathing was investigated as a possible way to improve cognitive performance [17]. Another examined the effect of 5 min of slow-paced breathing on cardiac vagal activity and revealed an increase in emotional arousal [18]. Brief slow-paced breathing has also been shown to improve immediate executive function [19]. Our results suggest that practicing breathing exercises briefly but regularly can improve bloating. Whether participants are able to continue practicing this long term and maintain the benefits remains to be investigated.

We did not find any difference between Calmigo^®^ and DB instructions. Both interventions significantly improved bloating compared to baseline, but no difference between groups was observed. This suggests that the therapeutic effect is likely due to practicing regular breathing exercises themselves, rather than the delivery method. The significance of our study lies in demonstrating that even simple, written instructions can be effective. This has important clinical implications, specifically in settings without access to specialized devices or biofeedback tools. Calmigo^®^ may still have value for patients who prefer structured feedback or struggle to follow self-guided breathing, but our results highlight that accessible, low-cost interventions can be equally beneficial.

We selected a 6-week active intervention with a 7th-week follow-up because most GI behavioral and lifestyle intervention studies are conducted over 6–8 weeks. This timeframe has been shown to be sufficient to observe symptom changes while maintaining feasibility and participant adherence [20,21]. The final 7th-week assessment allowed us to evaluate whether improvements persisted one week beyond the intervention.

Although our study is limited by the smaller sample size that completed the 6 weeks of breathing intervention, we observed a consistent improvement in bloating at each week of the study suggesting that the effect is consistent and not an artifact. Our study is practical because people may find it much easier to do a brief breathing exercise regimen regularly rather than longer periods, as suggested by other studies. Participants were encouraged to complete the brief training call on how to properly use the device, which may have also helped participants use Calmigo^®^ effectively. The fact that written instructions were effective in reducing bloating is encouraging, as many places may not have specialists who can teach people how to do diaphragmatic breathing.

## 5 Conclusions

Bloating is a common and often difficult problem to manage. Studies investigating the effect of breathing exercises on bloating are very limited. Our study found a significant and medium- to large-sized effect size on self-reported bloating with both written instructions for diaphragmatic breathing and a device that provides biofeedback during breathing. Baseline anxiety and depressive symptoms predict whether someone will experience an improvement in bloating. Future studies to examine the long-term effectiveness of breathing exercises on bloating and the underlying mechanisms remain will potentially pave the way for effective integration of breathwork in the management of those with bothersome bloating.

## Appendix 1

**Diaphragmatic Breathing**

**Diaphragmatic breathing technique**

1. Lie on your back on a flat surface or in bed, with your knees bent and your head supported. You can use a pillow under your knees to support your legs. Place one hand onyour upper chest and the other just below your rib cage. This will allow you to feel your diaphragm move as you breathe.
2. Breathe in slowly through your nose so that your stomach moves out against your hand. The hand on your chest should remain as still as possible.
3. Tighten your stomach muscles, letting them fall inward as you exhale through pursed lips. The hand on your upper chest must remain as still as possible.

When you first learn the diaphragmatic breathing technique, it may be easier for you to follow the instructions lying down, as shown on the first page. As you gain more practice, you can try the diaphragmatic breathing technique while sitting in a chair, as shown below.

**To perform this exercise while sitting in a chair:**

1. Sit comfortably, with your knees bent and your shoulders, head and neck relaxed.
2. Place one hand on your upper chest and the other just below your rib cage. This will allow you to feel your diaphragm move as you breathe.
3. Breathe in slowly through your nose so that your stomach moves out against your hand. The hand on your chest should remain as still as possible.
4. Tighten your stomach muscles, letting them fall inward as you exhale through pursed lips. The hand on your upper chest must remain as still as possible.

Note: You may notice an increased effort will be needed to use the diaphragm correctly. At first, you'll probably get tired while doing this exercise. But keep at it, because with continued practice, diaphragmatic breathing will become easy and automatic.

**How often should I practice this exercise?**

Practice this exercise 3 minutes 3 times per day.

**What is the Diaphragm?**

The diaphragm is a muscle that separate the organs in the chest from the abdominal organs.

**What is Diaphragmatic breathing?**

- It is a type of breathing that is seen in babies.
- In this type of breathing, a deep, full breath allows the lungs to fill with oxygen, moving the diaphragm downward and pushes the stomach out, to make room for all the air.
- This type of breathing increases the oxygen that enters the body, reducing tension.
- When you breathe out fully (stomach is tucked in), you also help to release all the tension in the body.
- When intentionally done, this type of breathing can slow your breathing rate, create a feeling of relaxation.
